# Intervertebral disc degeneration is rescued by TGFβ/BMP signaling modulation in an ex vivo filamin B mouse model

**DOI:** 10.1038/s41413-022-00200-5

**Published:** 2022-04-26

**Authors:** Jennifer Zieba, Kimberly N. Forlenza, Kelly Heard, Jorge H. Martin, Michaela Bosakova, Daniel H. Cohn, Stephen P. Robertson, Pavel Krejci, Deborah Krakow

**Affiliations:** 1 Department of Orthopedic Surgery, Los Angeles, CA 90095 USA; 2 Department of Biology, Faculty of Medicine, Masaryk University, 62500 Brno, Czech Republic; 3 International Clinical Research Center, St. Anne’s University Hospital, 65691 Brno, Czech Republic; 4 Institute of Animal Physiology and Genetics, Czech Academy of Sciences, 60200 Brno, Czech Republic; 5 Department of Molecular Cell and Developmental Biology, University of California, Los Angeles, Los Angeles, CA 90095 USA; 6 Department of Women’s and Children’s Health, Dunedin School of Medicine, University of Otago, Dunedin, New Zealand; 7 Department of Human Genetics, Los Angeles, CA 90095 USA; 8 Department of Obstetrics and Gynecology, Los Angeles, CA 90095 USA; 9 Department of Pediatrics, David Geffen School of Medicine at University of California, Los Angeles, Los Angeles, CA 90095 USA

**Keywords:** Physiology, Diseases

## Abstract

Spondylocarpotarsal syndrome (SCT) is a rare musculoskeletal disorder characterized by short stature and vertebral, carpal, and tarsal fusions resulting from biallelic nonsense mutations in the gene encoding filamin B (FLNB). Utilizing a FLNB knockout mouse, we showed that the vertebral fusions in SCT evolved from intervertebral disc (IVD) degeneration and ossification of the annulus fibrosus (AF), eventually leading to full trabecular bone formation. This resulted from alterations in the TGFβ/BMP signaling pathway that included increased canonical TGFβ and noncanonical BMP signaling. In this study, the role of FLNB in the TGFβ/BMP pathway was elucidated using in vitro, in vivo, and ex vivo treatment methodologies. The data demonstrated that FLNB interacts with inhibitory Smads 6 and 7 (i-Smads) to regulate TGFβ/BMP signaling and that loss of FLNB produces increased TGFβ receptor activity and decreased Smad 1 ubiquitination. Through the use of small molecule inhibitors in an ex vivo spine model, TGFβ/BMP signaling was modulated to design a targeted treatment for SCT and disc degeneration. Inhibition of canonical and noncanonical TGFβ/BMP pathway activity restored *Flnb*^−/−^ IVD morphology. These most effective improvements resulted from specific inhibition of TGFβ and p38 signaling activation. FLNB acts as a bridge for TGFβ/BMP signaling crosstalk through i-Smads and is key for the critical balance in TGFβ/BMP signaling that maintains the IVD. These findings further our understanding of IVD biology and reveal new molecular targets for disc degeneration as well as congenital vertebral fusion disorders.

## Introduction

Spondylocarpotarsal synostosis (SCT) is an autosomal recessive skeletal disorder characterized by carpal/tarsal bone and vertebrae fusions, delayed endochondral ossification, and short stature^1–6^. The majority of SCT cases result from biallelic nonsense mutations in the gene that encodes *filamin B* (*FLNB*)^2,5–7^. SCT is progressive; there is radiographic evidence of mild disc narrowing at birth ultimately advancing to severe spinal scoliosis, kyphosis, and lordosis due to premature vertebral fusions^1,2,5,6,8^.

Filamins are a family of large juxtamembrane cytoskeletal proteins that bind to and stabilize the three-dimensional actin network and serve as integrators of multiple signaling pathways, including TGFβ/BMP (reviewed in^9–13^). Canonical TGFβ/BMP signaling is initiated through TGFβ/BMP ligands that signal through varying combinations of type I and type II serine/threonine kinase receptors. Activation of the signaling cascade is evoked upon ligand binding and dimerization of the receptors. The intracellular domain of the receptor complex induces phosphorylation of receptor-regulated Smads (R-Smads): Smads 1, 5, and 8 or Smads 2 and 3 upon BMP or TGFβ ligand binding, respectively. These activated R-Smads subsequently form a complex with Smad 4, move into the nucleus, and regulate gene expression^14–18^. TGFβ and BMP ligand/receptor complexes can also activate noncanonical signaling pathways through the phosphorylation of extracellular-related kinase (ERK) or p38 mitogen-activated protein kinase^19–23^. Noncanonical pathway activation also allows signaling crosstalk between the two pathways through the phosphorylation of the Smad1 linker region by TGFβ-activated ERK^24,25^. Attenuation of the pathway through interactions with inhibitory Smads (i-Smads) allows for controlled intracellular regulation of signals. Further signaling control comes from phosphorylation of Smad linker regions, which induce R-Smad ubiquitination and subsequent degradation^26–30^. However, for R-Smads that are transported into the nucleus, phosphorylation of the linker region enhances their stability and increases nuclear target transcription and has recently become a focus as an independent signaling mechanism with many roles in disease^27,31,32^. Filamin A, a paralog of FLNB, interacts with Smads; in HEK293 cells, filamin A (FLNA) interactions with Smads 1, 2, 4, 5 and 6 were established and have recently been reported to regulate the balance between ERK and Smad activation^33,34^. FLNB has been shown to interact with Smad 3, but other Smad interactions have not been explored^33,35^.

Disc degeneration is a progressive aging disease affecting greater than one-third of the adult population.^36^ Proposed mechanisms include IVD degeneration due to cell loss, inflammatory responses, and increased extracellular matrix (ECM) degradation coupled with reduced synthesis, eventually leading to mechanical collapse^37–41^. TGFβ/BMP signaling pathways have been implicated in IVD development, homeostasis, and degeneration. Previous work showed increases in both BMP and TGFβ signaling in aging disc degeneration, although inconsistencies exist regarding whether modulation of TGFβ/BMP signaling restored the IVD or accelerated ectopic ossification of the IVD tissues^38,41–45^. Further supporting the role of altered TGFβ signaling in disc degeneration, patients with mutations in the gene encoding *SMAD3* resulting in increased Smad3 phosphorylation showed disc degeneration beginning as early as 12 years of age^45^. Myhre syndrome, an autosomal dominant disorder due to heterozygosity for mutations in the gene encoding *SMAD4*, is also associated with premature vertebral fusions^46–48^. Furthermore, we previously demonstrated that in the IVD of the *Flnb* knockout mouse model, there was enhanced TGFβ and BMP pathway activity, specifically increased levels of phosphorylated Smad 3 (p-Smad3), p-p38, p-ERK, and p-Smad 1. These changes contributed to abnormal signaling and morphologic changes producing premature disc degeneration^49,50^.

In this study, we further elucidated the function of FLNB in the IVD and established its role in TGFβ/BMP crosstalk by showing that FLNB functions as a negative regulator of the TGFβ/BMP pathway. It does so by interacting with and stabilizing i-Smads and ultimately allowing for proper targeting and ubiquitination of R-Smads as a means to attenuate signaling. Using a spine organ culture model treated with specific TGFβ and BMP inhibitors and activators, we determined that the abnormal IVD phenotype could be induced and modulated. Inhibition of TGFβ signaling through Smad 2/3, ERK, and p38 activation effectively rescued the abnormal phenotype found in the *Flnb*^−/−^ IVD. This work delineates the mechanism by which FLNB modulates canonical and noncanonical TGFβ/BMP signaling through i-Smads and provides a targeted molecular model to understand progressive disc degeneration.

## Results

### *Flnb*^−/−^ IVDs undergo mineralization and trabecular bone formation

**Fig. 1:**
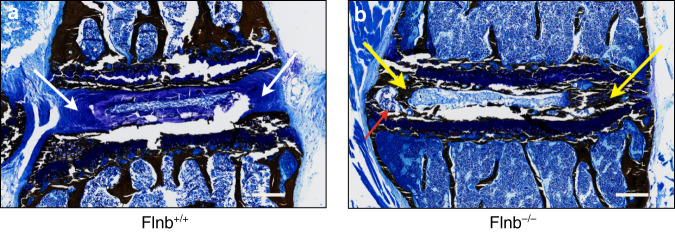
*Flnb*^−/−^ IVD exhibits ectopic mineralization. **a**, **b** P21 IVD plastic sagittal sections with Von Kossa staining. **a** *Flnb*^+/+^ IVD exhibiting blue–violet fibrocartilage in AF IVD tissue (white arrows). **b** *Flnb*^−/−^ IVD exhibits black–brown staining in the AF representing mineralization of the AF tissue (yellow arrows). AF also exhibits the characteristics of trabecular bone, an indication that it is undergoing full ossification. *n* = 3, Representative image of T7-T8 IVD in individual mice

Previous work showed that in the absence of FLNB, the extracellular matrix (ECM) of the annulus fibrosus (AF) of the IVD undergoes changes from fibrous type I collagen-expressing tissue to a cartilaginous type II collagen matrix^50^. By postnatal Day 15 (P15), the ECM had progressively increased cartilaginous proteoglycan secretion throughout the collapsing disc. Histologic analyses showed that fibroblastic-like AF cells underwent cartilaginous hypertrophy, as demonstrated by morphologic changes, ectopic secretion of type X collagen, and absence of the disc between the vertebral bodies^50^. To detail the abnormal mineralization process in the disc, *Flnb*^−/−^ P21 mouse spine tissues were embedded in plastic for serial sectioning. Von Kossa with MacNeal’s tetrachrome staining enabled visualization of the mineralization pattern within *Flnb*^+/+^ and *Flnb*^−/−^ IVDs. While the *Flnb*^+/+^ AF tissues exhibited the blue–violet staining expected for fibrocartilage (Fig. 1a, white arrows), the *Flnb*^−/−^ IVDs showed clear mineralization in the form of black staining throughout the AF tissue (Fig. 1b, yellow arrows) as well as the beginnings of trabecular bone formation indicated by the beginnings of bone marrow formation within the AF area (Fig. 1b, red arrow). This indicated that the AF tissue underwent bony mineralization, promoting the development of vertebral body fusions.

### Isolated *Flnb*^−/−^ AF cells showed increased TGFβ and BMP signaling

**Fig. 2:**
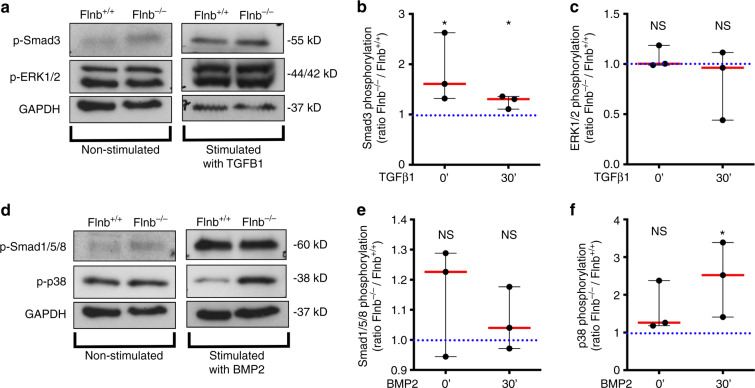
Isolated primary *Flnb*^−/−^ AF cells exhibit increased TGFβ, ERK, and p38 signaling activation. **a** Western blots of protein lysates isolated from unstimulated primary AF cells (left column) and stimulated with 5 ng·mL^−1^ TGFβ-1 ligand for 30 min (right column) probed with antibodies against p-Smad 3, p-ERK1/2, and GAPDH. **b** Quantification of TGFβ-1-stimulated Western blot normalized against GAPDH levels showing a significant increase in Smad 3 phosphorylation in both unstimulated and stimulated primary AF cells. **c** Quantification of TGFβ-1-stimulated Western blot normalized against GAPDH levels showing no significant increase in ERK1/2 phosphorylation in either unstimulated or stimulated primary AF cells. **d** Western blots of isolated unstimulated primary AF cells (left column) and stimulated with 5 ng·mL^−1^ BMP-2 ligand for 30 min (right column) probed with antibodies against p-Smad 1/5/8, p-p38, and GAPDH. **e** Quantification of BMP-2-stimulated Western blot normalized against GAPDH levels showing no significant increase in Smad 1/5/8 phosphorylation. **F** Quantification of BMP-2-stimulated Western blot normalized against GAPDH levels showing a significant increase in p38 phosphorylation in both unstimulated and stimulated primary AF cells. *n* = 9 (each data point contains pooled IVD tissue samples from three mice containing six IVDs from each mouse; data points represent biological replicates of *Flnb*^−/−^ protein lysates each normalized to a *Flnb*^+/+^ control. The dotted blue line in each graph represents the *Flnb*^+/+^ protein level, which is set to 1 upon normalization. Significance is determined by comparing *Flnb*^−/−^ to *Flnb*^+/+^), NS not significant, *P < 0.05, **P < 0.01, ***P < 0.001

In primary chondrocytes and whole IVD lysates, both canonical and noncanonical TGFβ signaling was increased in the absence of FLNB, whereas noncanonical BMP signaling was increased while canonical BMP signaling remained unchanged^50^. To confirm the aforementioned findings directly in AF cells, IVDs were dissected, the nucleus pulposus (NP) was removed, and AF cells were isolated. Cells were serum starved overnight and then stimulated with TGFβ-1 or BMP-2 ligands for 30 min. Although no change in ERK phosphorylation was detected, which was previously seen in both chondrocytes and whole IVD lysates, there was an increase in Smad3 phosphorylation in *Flnb*^−/−^ cells both endogenously and after TGFβ-1 stimulation (Fig. 2a–c). In a previous study, an increase in ERK phosphorylation was observed at the 10-minute stimulation time point. Because of cell number constraints and the desire to reduce the number of animals used in the study, 10-minute time points were not performed using AF cells, perhaps explaining the lack of observed increased ERK phosphorylation. The previously observed increase in p38 phosphorylation after BMP stimulation and no change in the levels of Smad 1/5/8 phosphorylation between WT and *Flnb*^−/−^ cells were confirmed (Fig. 2d–f). Similar to liberated chondrocytes and whole tissue IVDs, the more relevant AF cells demonstrated altered TGFβ canonical and BMP noncanonical signaling pathways in the absence of FLNB.

### Loss of FLNB leads to persistent Smad 1 activity through linker phosphorylation and decreased ubiquitination

**Fig. 3:**
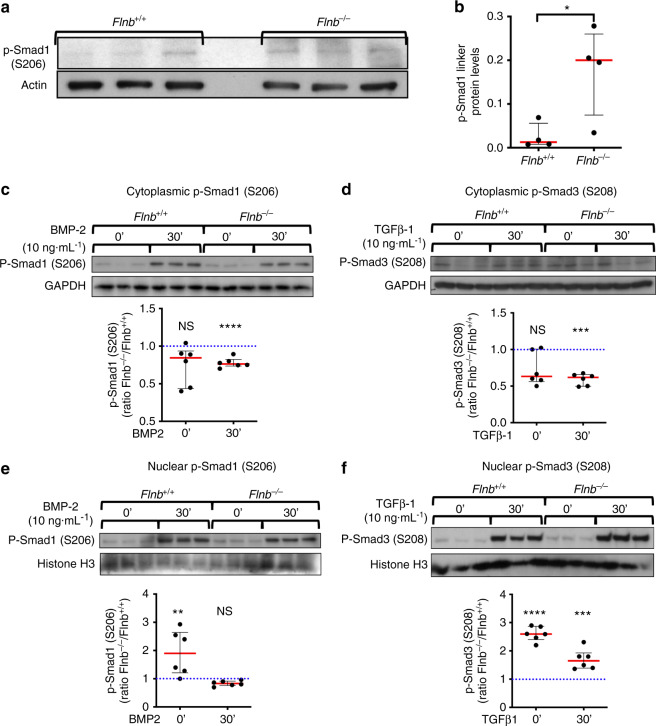
Higher levels of linker phosphorylated Smads 1 and 3 were found in the nucleus of *Flnb*^*−*^^/−^ primary chondrocytes. **a** Western blot of Smad 1 linker phosphorylation levels in protein lysates isolated from *Flnb*^+/+^ and *Flnb*^−/−^ IVDs. **b** Quantification of Western blot normalized to Actin levels showing a significant increase in Smad 1 linker phosphorylation. *n* = 3 (each data point contains six IVDs dissected from the thoracic area from a single mouse), NS not significant, *P value < 0.05. **c**–**f** Above: Western blots of *Flnb*^+/+^ and *Flnb*^−/−^ cytoplasmic and nuclear protein lysate fractions isolated from primary sternal chondrocytes unstimulated and stimulated with 5 ng·mL^−1^ TGFβ-1 ligand and 10 ng·mL^−1^ BMP-2 ligand for 30 min probed with antibodies against Smad 3 linker phosphorylation at the S208 residue, Smad 1 linker phosphorylation at the S206 residue, GAPDH, and histone H3. Below: Quantification of TGFβ-1- and BMP-2-stimulated Western blots normalized against GAPDH (cytoplasmic fractions) and histone H3 (nuclear fractions) levels. The results showed a significant decrease in linker phosphorylated Smad 3 and Smad 1 levels in cytoplasmic fractions but a significant increase in nuclear fractions of *Flnb*^−/−^ primary chondrocytes. *n* = 6 (All data points represent biological replicates of *Flnb*^−/−^ protein lysates, each normalized to a *Flnb*^+/+^ control. The dotted blue line in each graph represents the *Flnb*^+/+^ protein level, which is set to 1 upon normalization. Significance is determined by comparing *Flnb*^−/−^ to *Flnb*^+/+^), NS not significant, *P < 0.05, ** *P* < 0.01, ***P < 0.001

Levels of phosphorylation of R-Smad proteins are used as a measure of activation of the TGFβ/BMP pathways. Our previous work showed no change in BMP-induced receptor phosphorylation of Smad 1/5/8 in *Flnb*^−/−^ chondrocytes and whole IVD lysates but showed an increase in Smad 1 nuclear target transcription^50^. Therefore, the molecular mechanism underlying this paradox was explored. All R-Smads contain phosphorylation sites within their linker regions that act as additional regulatory mechanisms, particularly to attenuate signals. The linker regions of Smad 2/3 (Serine208) and Smad 1/5/8 (Serine206) are phosphorylated by ERK, and when this occurs within the cytoplasm, Smads are bound by Smurfs 1 and/or 2 and subsequently targeted for ubiquitination^29,31,51,52^. However, if linker phosphorylated Smads avoid ubiquitination and are transported to the nucleus, phosphorylation of the linker site can increase the transcription of Smad nuclear targets through increased protein stabilization and nuclear binding^31^. Therefore, we hypothesized that in the absence of FLNB, increased Smad 1 nuclear target transcription occurred as a consequence of changes in linker region phosphorylation. Extracted proteins from *Flnb*^+/+^ and *Flnb*^−/−^ IVD whole lysates were probed for Smad 1 linker phosphorylation and showed significantly increased phosphorylation in *Flnb*^−/−^ IVDs (Fig. 3a, b). To determine whether the increase in Smad linker phosphorylated protein levels was localized to the nucleus versus the cytoplasm, cell fractionation experiments were performed for cytoplasmic and nuclear fractions of both linker phosphorylated Smad 1 (BMP signaling) and Smad 3 (TGFβ signaling). Isolating primary AF cells resulted in cell counts far too low for these in vitro experiments; therefore, *Flnb*^+/+^ and *Flnb*^−/−^ primary sternal chondrocytes were stimulated with 5 ng·mL^−1^ BMP-2 or 5 ng·mL^−1^ TGFβ-1. Endogenously, we found higher levels of Smad 1 and Smad 3 linker phosphorylation in the nuclei of *Flnb*^−/−^ primary chondrocytes *than in Flnb*^*+/+*^ primary chondrocytes (Fig. 3e, f). While endogenous cytoplasmic levels remained unchanged, lower levels of Smad 1 and Smad 3 linker phosphorylation were seen in the cytoplasm following 30 min of BMP stimulation (Fig. 3c, d). These data confirmed previous findings, shown by immunofluorescence staining, of overall increased receptor phosphorylated Smad 1 and Smad 3 localization in the nuclei of *Flnb*^−/−^ chondrocytes. Furthermore, this indicated that, in the absence of FLNB, increased levels of linker phosphorylated Smads are translocated into the nucleus and likely activate nuclear target transcription.

**Fig. 4:**
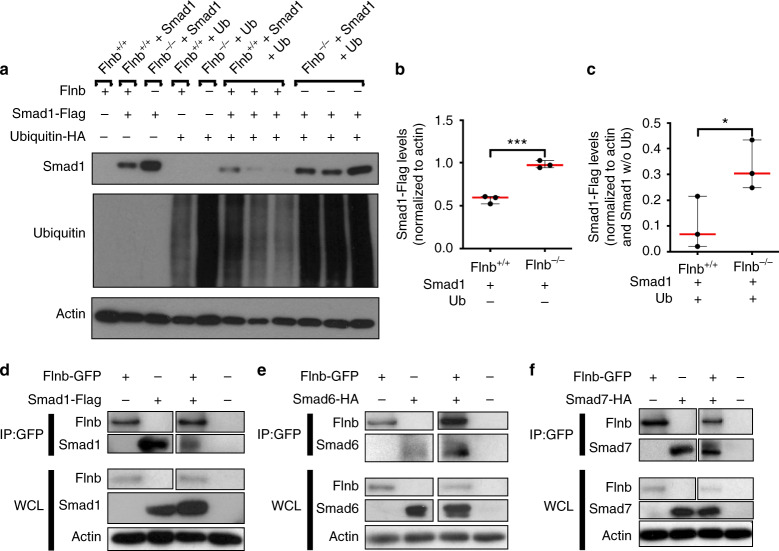
Smad 1 degradation is less efficient in *Flnb*^−/−^ -transfected MEFs due to FLNB interaction with i-Smads. **a** Western blots of *Flnb*^+/+^ and *Flnb*^*–/–*^ MEF lysates nontransfected or transfected with Smad 1-Flag plasmid, ubiquitin-HA plasmid, or both. Blots were probed using antibodies against Flag, HA, and Actin. **b** Quantification of total Smad 1-Flag levels in *Flnb*^+/+^ versus *Flnb*^*–/–*^ MEFs normalized to actin levels. The results show that more Smad 1-Flag plasmid remained after transfection and that the 24-h recovery period indicated inefficient degradation in the absence of FLNB. **c** Quantification of total Smad 1-Flag levels in *Flnb*^+/+^ versus *Flnb*^*–/–*^ MEFs transfected with ubiquitin normalized to both Actin levels and Smad 1-Flag levels without ubiquitin transfection. The results again show that more Smad 1-Flag plasmid remained after ubiquitin transfection, indicating a lower efficiency in ubiquitinated degradation in the absence of FLNB. *n* = 3, *P < 0.05, ***P < 0.001. **d** Western blots of lysates isolated from wild-type MEFs transfected with FLNB-GFP and Smad 1-Flag plasmids immunoprecipitated using an antibody against GFP. FLNB western blot was probed using a GFP antibody, and Smad 1 western blot was probed using a Flag antibody. The results showed the detection of Smad 1-Flag protein when lysates were immunoprecipitated using a GFP antibody, confirming the in vitro interaction of FLNB and Smad 1. **e** Western blots of lysates isolated from wild-type MEFs transfected with FLNB-GFP and Smad 6-HA plasmids immunoprecipitated using an antibody against GFP. The results showed the detection of Smad 6-HA protein when lysates were immunoprecipitated using a GFP antibody, confirming the in vitro interaction of FLNB and Smad 6. **f** Western blots of lysates isolated from wild-type MEFs transfected with FLNB-GFP and Smad 7-HA plasmids immunoprecipitated using an antibody against GFP. The results showed the detection of Smad 7-HA protein when lysates were immunoprecipitated using a GFP antibody, confirming the in vitro interaction of FLNB and Smad 7. *n* = 3, WCL Whole Cell Lysate, IP Immunoprecipitate

Cytoplasmic linker phosphorylated R-Smads are typically targeted for degradation via the ubiquitin pathway as a means of regulating signal strength. Because of increased endogenous levels of linker phosphorylated R-Smads within the nucleus, we hypothesized that absence of FLNB altered proper ubiquitination of R-Smads, thus allowing for enhanced availability of R-Smads traveling to the nucleus. To investigate this possibility, we transfected *Flnb*^−/−^ and *Flnb*^+/+^ mouse embryonic fibroblasts (MEFs) with a Flag-tagged Smad 1 and HA-tagged ubiquitin plasmid via electroporation. In *Flnb*^−/−^ MEFs transfected with Smad 1, there was an increase in overall Smad 1 levels when compared with *Flnb*^+/+^ MEFs (Fig. 4a, b). In cells transfected with both Smad 1 and ubiquitin, there were decreased Smad 1 levels in both *Flnb*^−/−^ and *Flnb*^+/+^. However, the decrease in Smad 1 levels in *Flnb*^−/−^ MEFs was significantly less than that in *Flnb*^+/+^ (Fig. 4a, c). Therefore, in the absence of FLNB, Smad 1 was degraded less efficiently. This indicates that wild-type FLNB plays a role in the cytoplasmic retention and eventual ubiquitination of R-Smad proteins. Overall ubiquitin levels were also consistently higher in *Flnb*^−/−^ mice, indicating a possible role for FLNB in ubiquitin turnover (Fig. 4a).

### FLNB interacted with inhibitory Smads (i-Smads)

The paralogous protein FLNA has been shown to interact with several Smads, although this has not been demonstrated for FLNB^33^. Smad 4 functions as a co-Smad, forming a complex with activated receptor Smads and aiding in nuclear translocation^17^. Smad 6 functions as an i-Smad and inhibits both p38 activation and Smad 1 translocation into the nucleus without inhibiting receptor-mediated phosphorylation by competing with Smad 4 binding^26^. Smad 7 also functions in signaling inhibition by competing for TGFβ receptor phosphorylation of R-Smads 2 and 3^53,54^. We tested the hypothesis that FLNB interacts with these i-Smads, facilitating the formation of complexes with TGFβ receptors or Smad 1 by acting as a protein complex-stabilizing molecule. Interactions between FLNB and i-Smads were examined in MEFs by coimmunoprecipitation experiments using GFP-tagged human FLNB and HA or Flag-tagged human Smad plasmids through transfection via electroporation. Using GFP pull-down, FLNB showed interactions with receptor Smad 1 and i-Smads 6 and 7 (Fig. 4d–f). These findings demonstrated that FLNB interacts with i-Smads, supporting FLNB’s role in TGFβ/BMP signaling attenuation.

### Small molecule inhibition of TGFβ signaling and p38 activation restored *Flnb*^−/−^ IVD tissue architecture and ECM composition

Increases in TGFβ/BMP signaling due to loss of FLNB and the underlying cellular mechanisms were established through in vitro experiments and in vivo characterization of *Flnb*^−/−^ mice. We sought to determine whether the pathology can be recapitulated using an organ culture of the thoracic spine based on previously published methods, thereby generating a model that can be more easily manipulated^55,56^. Dissected P1 thoracic spines were cultured and treated for two weeks, after which paraffin sections were generated. One of the main hallmarks of disc degeneration is changes in the ECM composition of the AF. Changes in proteoglycan secretion and collagen composition are used as identifiers of progressive degeneration (reviewed in^37,57^). We performed alcian blue staining and IHC for type II collagen localization to detect whether the treatments had an effect on the composition of the ECM and, indirectly, the integrity of the IVD itself.

**Fig. 5:**
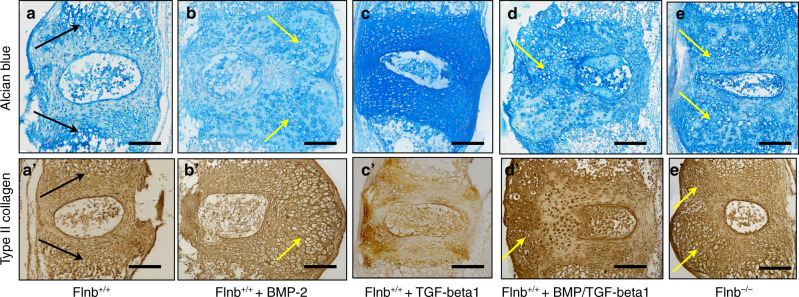
*Flnb*^+/+^ IVD stimulation with TGFβ-1 and BMP-2 mimics the Flnb^–/–^ phenotype, which is rescued by combinatorial inhibition of TGFβ and p38 signaling. **a**–**e** Representative images of sagittal sections of T7-T8 cultured IVDs stained with Alcian Blue for proteoglycans. **a** *Flnb*^+/+^ cultured spines show proteoglycans concentrated in the articular cartilage (black arrows). **b** BMP-2-stimulated *Flnb*^+/+^ spines exhibited no change in proteoglycan secretion but severe disruptions in tissue architecture (yellow arrows). **c** TGFβ-1-stimulated *Flnb*^+/+^ spines showed no change in tissue architecture but increased proteoglycan secretion. **d** BMP-2- and TGFβ-1-stimulated *Flnb*^+/+^ spines exhibit increased proteoglycan secretion and disruptions in tissue architecture (yellow arrow). **e** *Flnb*^*–/–*^spine exhibiting increased proteoglycan secretion and disrupted tissue architecture (yellow arrows). **a’**–**e’** Representative images of sagittal sections of T7-T8 cultured IVDs stained for type II collagen via IHC-HRP. **a’** Type II collagen distribution is more evenly distributed throughout the IVD concentrated in the AC. **b’** BMP-2-stimulated *Flnb*^+/+^ spines exhibit increased type II collagen secretion and severe disruptions in tissue architecture (yellow arrow). (C’) TGFβ-1 stimulated *Flnb*^+/+^ spines, showing no change in tissue architecture or type II collagen secretion. **d’** BMP-2- and TGFβ-1-stimulated *Flnb*^+/+^ spines exhibit increased type II collagen and tissue architecture disruptions (yellow arrow). **e’** *Flnb*^*–/–*^ cultured spines exhibiting increased type II collagen secretion and disrupted tissue architecture (yellow arrows)

In previous work, *Flnb*^−/−^ IVDs exhibited increased proteoglycan secretion and type II collagen in AF tissues, whereas these proteins were normally localized to the endplate regions in *Flnb*^+/+^ IVDs^50^. After two weeks in culture, *Flnb*^+/+^ cultured spines showed a low level of proteoglycan and type II collagen secretion, and as expected, proteoglycan and type II collagen distribution were mainly concentrated near the endplate cartilage areas (Fig. 5a, a’ arrows). *Flnb*^−/−^ cultured spines, however, displayed a high level of proteoglycan and type II collagen secretion that was widely dispersed throughout the AF (Fig. 5e, e’). Furthermore, the tissue architecture of *Flnb*^−/−^ cultured spines was highly disrupted, exhibiting expanded, rounded cells, overall tissue expansion, and NP disruption when compared with *Flnb*^+/+^ discs (Fig. 5e, e’, arrows). Overall, the ECM changes exhibited in *Flnb*^−/−^ cultured spines reflected those found in in vivo P1 *Flnb*^−/−^ spine sections supporting the spine culture model.

To confirm that the increase in TGFβ/BMP signaling underlie these changes in matrix composition within this in vitro model, *Flnb*^+/+^ spines were stimulated with TGFβ-1 and BMP-2 ligands over a two-week time period. *Flnb*^+/+^ spines treated with BMP2 showed a change in tissue architecture in the form of expanded tissue area and the presence of hypertrophic-like cells (yellow arrows), no change in overall proteoglycan level appearance, but an increase in type II collagen secretion (Fig. 5b, b’). Conversely, when treated with TGFβ-1, *Flnb*^+/+^ spines showed an increase in proteoglycan levels that was dispersed throughout the disc but no change in type II collagen secretion or disc architecture (Fig. 5c, c’). This suggested that both the BMP and TGFβ pathways have distinct roles in postnatal IVD maintenance. When *Flnb*^+/+^ spines were treated simultaneously with both BMP2 and TGFβ-1, there was an increase in proteoglycan levels, dispersed proteoglycan distribution, increased type II collagen secretion, and a disruption in disc architecture (Fig. 5d, d’, arrows). These effects mimic the cultured *Flnb*^−/−^ spine findings and support the biochemical findings that showed that increased TGFβ and BMP signaling contributed to the phenotypic abnormalities. This also indicated that under normal conditions, the modulation of TGFβ/BMP signaling plays an important role in the maintenance of disc composition and integrity. Additionally, after two weeks in culture, *Flnb*^−/−^ spines did not exhibit disc narrowing/collapse, which is usually seen in vivo, suggesting that mechanical forces play an additional role in the initial collapse of the *Flnb*^−/−^ disc (Fig. 5e, e’).

To further confirm our in vitro findings and potentially target components of the TGFβ and BMP pathways for treatment, cultured *Flnb*^−/−^ spines were continually exposed to various inhibitors during their two-week culture, and then proteoglycan and type II collagen levels were examined using *Flnb*^+/+^ spines as controls. Small molecule and protein inhibitors as well as their respective concentrations used are summarized in Table 1. Individually, the inhibitors had varying effects on proteoglycan secretion and tissue architecture (Fig. S1). Treatment of spines with the Noggin protein inhibitor, an inhibitor of BMP receptor activation, reduced proteoglycan levels in *Flnb*^−/−^ discs but did not restore architecture or type II collagen levels (Fig. S1b, b’, b”). TGFβ inhibition via small molecule SB-431542 (an inhibitor of Smad 2/3 phosphorylation) and SB-505124 (an inhibitor of Smad 2/3 and ERK phosphorylation) treatment caused severe disruptions in tissue architecture in *Flnb*^−/−^ discs (yellow arrows) (Fig. S1c, c’, c”, d, d’, d”). The individual inhibition of ERK via the small molecule inhibitor U0126 (an inhibitor of ERK phosphorylation via MEK inactivation) increased proteoglycan levels in *Flnb*^−/−^ discs but did restore some architectural abnormalities (Fig. S1e, e’, e”). Finally, treatment with a small molecule inhibitor of p38 phosphorylation (SB-203580) alone rescued tissue architecture as well as type II collagen levels and distribution in *Flnb*^−/−^ discs, although it did not reduce proteoglycan levels (Fig. S1f, f’, f”). These results show that while there may have been evidence for rescued or improved phenotype, modulation of a single pathway or pathway component is not sufficient to rescue the *Flnb*^−/−^ pathology.

**Table 1:** Protein and small molecule inhibitors used in cultured spine treatments

Inhibitor	Catalog number	Inhibition activity	Conc. used	Reference
Noggin	Sigma SRP4675	BMP ligand binding by binding directly to multiple BMP ligands	100 ng·mL^−1^	^82^
431542	Sigma S4317	Inhibits Smad 2/3 phosphorylation through kinase activity inhibition of Alk4, Alk5, and Alk7	100 μmol·L^−1^	^83^
505124	Sigma S4696	Inhibits Smad2/3 and ERK phosphorylation through kinase activity inhibition of Alk4, Alk5, and Alk7 more potently than 431542	50 μmol·L^−1^	^84^
U0126	Sigma U120	Inhibits kinase activity of Mek1 and Mek2, inhibits ERK phosphorylation but not ERK kinase activity	250 μmol·L^−1^	^85^
203580	Millipore 559389	Binds to p38 and competes for ATP, inhibiting kinase activity	100 μmol·L^−1^	^86,87^

**Fig. 6:**
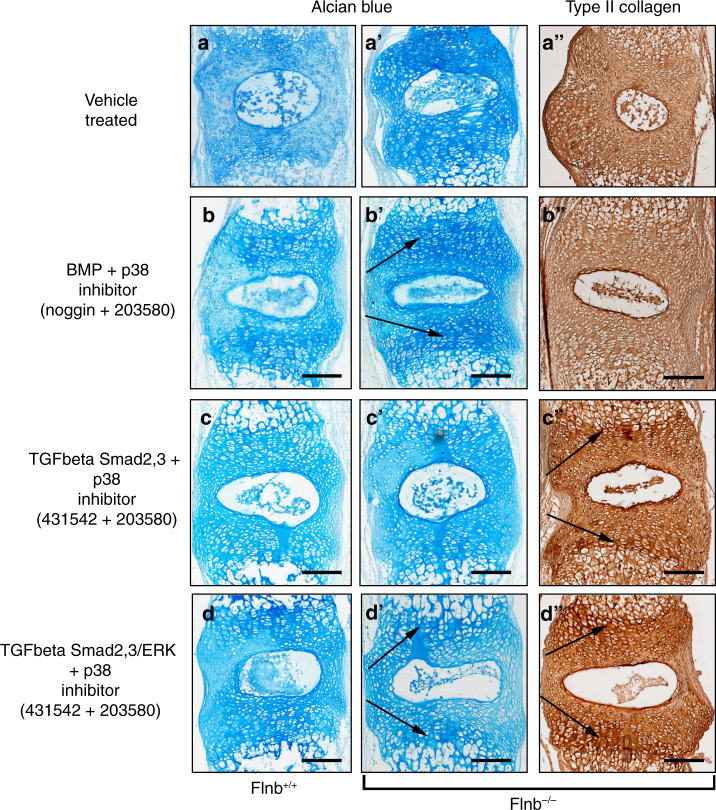
The *Flnb*^−/−^ phenotype is rescued by combinatorial inhibition of TGFβ and p38 signaling. **a**–**d** Representative images of sagittal sections of T7-T8 cultured IVDs stained with Alcian Blue for proteoglycan secretion or IHC-HRP using an antibody against type II collagen. **a**–**a”** *Flnb*^*–/–*^spine exhibiting increased proteoglycan secretion Type II Collagen secretion and disrupted tissue architecture (**b**–**b”**) Inhibition of BMP/p38 in combination reduces proteoglycan secretion in the AF while maintaining higher levels in the AC of *Flnb*^*–/–*^ IVDs and shows improvement of Type II Collagen levels but not distribution (black arrows). **c**–**c”**) Inhibition of TGFβ/Smad2,3/p38 improved proteoglycan levels but not distribution while reducing type II collagen secretion in the AF and maintaining higher levels in the AC of *Flnb*^*–/–*^ IVDs (black arrows). **d**–**d”** Inhibition of TGFβ/Smad2,3/ERK/p38 in combination reduces proteoglycan secretion, and type II collagen in the AF maintains higher levels in the AC of *Flnb*^*–/–*^ IVDs (black arrows) while also rescuing tissue architecture. *n* = 3 individual mouse spines with multiple affected IVDs

*Flnb*^+/+^ and *Flnb*^−/−^ cultured spines were therefore exposed to combinations of inhibitors in an effort to inhibit the same pathway components that were demonstrated to be increased via molecular experiments. As a comparison, *Flnb*^*+/+*^ spines showed low levels of proteoglycans and type II collagen secretion in mainly concentrated near the endplate cartilage areas with minimal tissue disruption after two weeks in culture whereas *Flnb*^*–/–*^ spines exhibited increased levels and significant tissue abnormalities (Fig. 6a, a’, a”). Several combinations did not result in rescue of proteoglycan and type II collagen levels or tissue architecture and are reported in Fig. S2. The inhibition of the BMP pathway (via Noggin) and p38 activation (via SB 203580) reduced proteoglycan secretion/distribution and repaired tissue architecture (Fig. 6b, b’, b”) when compared to vehicle treated Flnb^–/–^ spines showing increased proteoglycan secretion and tissue architecture disruption (Fig. 6a’, a”). This treatment combination, however, only mildly improved type II collagen levels and did not improve type II collagen distribution, indicating that inhibition of BMP at the receptor level does not sufficiently rescue the *Flnb*^−/−^ IVD (Fig. 5f”). Cultured *Flnb*^−/−^ spines exposed to TGFβ/Smad 2/3 (via SB-431542) and p38 inhibitors exhibited repaired disc architecture, improved type II collagen levels/distribution, and lowered proteoglycan levels but did not improve proteoglycan distribution when compared to untreated *Flnb*^−/−^ spines (Fig. 6c, c’, c”). Ultimately, based on improved cellular appearance and reduced proteoglycan and type II collagen expression, TGFβ/Smad 2/3, ERK and p38 inhibition was found to be most effective in rescuing the *Flnb*^−/−^ IVD phenotype (Fig. 6d, d’, d”). These results suggest that although we did not observe increased levels of ERK phosphorylation in our cultured AF cell experiments, ERK overactivation remains relevant and that inhibition of these specific pathway components effectively repairs IVD ECM composition and structure in *Flnb*^−/−^ cultured spines.

## Discussion

**Fig. 7:**
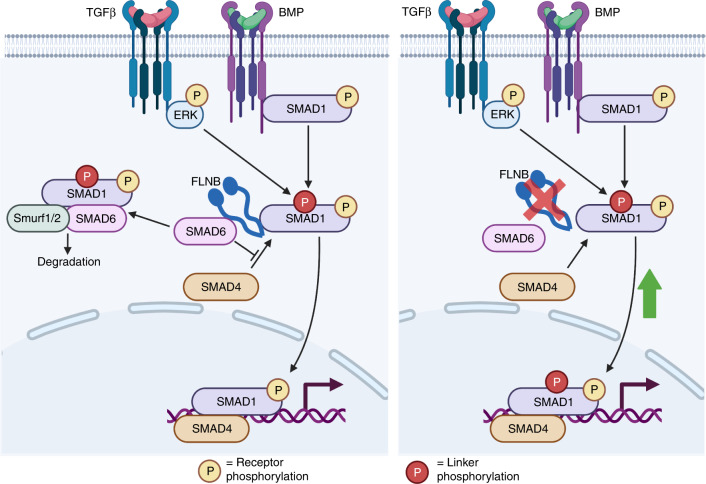
FLNB stabilizes i-Smad and Smad1 binding, inducing Smad1 degradation. Our model illustrates that under wild-type conditions (**a**), the Smad1 linker region is phosphorylated by TGFβ-activated ERK, which tags the molecule for degradation through the recruitment of Smad6, whose interaction with Smad1 is stabilized by FLNB. In the absence of FLNB (**b**), linker phosphorylated Smad1 is degraded less efficiently and allowed to induce increased transcription of nuclear targets

Vertebral fusions in the *Flnb*^−/−^ mouse, a model of SCT, result from degeneration, collapse, and ossification of the IVD. Our previous studies showed that TGFβ/BMP signaling was upregulated in *Flnb*^−/−^ chondrocytes and whole lysate IVDs and was likely the cause of ectopic IVD ossification, but the mechanism by which this occurred remained unresolved. Using primary AF cell culture and spine organ culture methods, we confirmed that the degeneration and ossification of the *Flnb*^−/−^ IVD was instigated by increased TGFβ/BMP signaling and that inhibition of specific TGFβ/BMP pathway components prevented the degeneration of *Flnb*^−/−^ IVDs. These signaling alterations resulted from the loss of normal FLNB interactions with i-Smad 6 and 7. Although we observed no change in the receptor phosphorylation of Smad 1 in primary AF cells, there was increased Smad 1 nuclear localization. Our previous work also showed increased Smad 1 nuclear target transcription, indicating that Smad 1 is transported into the nucleus more efficiently in the absence of FLNB^50^. Our current data demonstrated that in the absence of FLNB, appropriate cytoplasmic Smad 1 degradation is diminished. Ubiquitination of Smads is an important regulatory mechanism in both the TGFβ and BMP pathways to attenuate signaling^58,59^. Ubiquitination of Smad 1 results from the binding of i-Smad 6, which then attracts the binding of E3 ubiquitin ligase Smurf1 and Runx2, resulting in the degradation of Smad 1^26–28,30^. We showed that FLNB interacted with i-Smad 6 and contributed to the regulation of Smad 1 activity, a regulatory mechanism that has not been previously characterized. Loss of FLNB resulted in a decrease in Smad 1 degradation by the ubiquitin pathway. Therefore, FLNB acts as a scaffold for Smad 1/Smad 6 complex formation, and without it, Smad 1 is free to move into the nucleus and activate transcription (Fig. 7). Smad 6 knockout mice exhibit defects in axial and appendicular skeletal development in the form of shortened long bones, sternal fusions, rounded IVDs, and an increase in BMP signaling^60^. This phenotypic and biochemical overlapping finding supports that in *Flnb*^−/−^ mice, Smad 6 fails to function properly^49^. Interestingly, *Flnb*^−/−^ cells consistently showed higher overall ubiquitination levels, even though we still observed reduced degradation of Smad1 (Fig. 4a). This could indicate a reduction in ubiquitin turnover as a result of the absence of FLNB and requires further study.

We also demonstrated the novel in vitro interaction of FLNB with Smad 7. Smad 7 knockout mice also exhibited skeletal defects, including shortened long bones and sternal fusions, analogous to *Flnb*^−/−^ mice^61^. Similar to Smad 6, Smad 7 also competes with Smad 4 binding of Smad 1 or Smad 3 to induce ubiquitination and attenuate target gene transcription.^62^ The findings support that FLNB stabilizes the Smad 1/Smad 7 and Smad 3/Smad 7 complex and that in the absence of FLNB, the R-Smads are able to freely move into the nucleus. However, our data have repeatedly demonstrated an increase in the receptor phosphorylation of Smad 3 but not Smad 1. This discrepancy may result from Smad 7 also binding to TGFβ/BMP type I receptors and targeting them for ubiquitination^63,64^. FLNB may stabilize the TGFB receptor/Smad 7 complex but not the BMP receptor/Smad 7 complex, and thus Smad3, but not Smad1 phosphorylation, would be influenced.

Disc degeneration is characterized by changes in the structure and composition of the ECM^37,57^, and similarities observed between IVDs in our *Flnb*^−/−^ mice and degenerative disc disease (DDD) patients provide a unique opportunity to test therapeutics that are based on molecular mechanism(s)^50^. Past treatments for DDD include removal of the entire disc or replacement of the nucleus pulposus (NP) (reviewed in^65,66^). However, these treatments were not entirely effective^67^. More recent treatment advances involve TGFβ/BMP and growth factor biologic therapies aimed at increasing anabolic activity and restoring the ECM within the AF (reviewed in^66,68,69^). However, this approach results in excessive proteoglycan secretion and adversely induces ectopic ossification of the AF tissue^43,44^. Interestingly, in degenerative disc disease, increased TGFβ activity was observed through IHC staining of diseased AF tissues, and this was interpreted as a response to tissue degeneration^70^. However, our in vivo and in vitro findings showed that increased TGFβ and BMP pathway activities produced increased proteoglycan and type II collagen secretion because normal fibroblast-like AF cells develop inappropriate chondrocytic behavior, instigate chondrification of AF cells, endochondral ossification of the disc, and ultimately degeneration. Therefore, while anabolic ligand therapies could restore some ECM properties, they may also induce damaging cell fate changes within the postnatal IVD.

Although alterations in multiple components of the TGFβ and BMP pathways were observed in our model, developing therapeutic targets requires determining the extent and specificity of the signaling components that affect the postnatal IVD. The simultaneous inhibition of TGFβ and p38 signaling resulted in the most effective rescue of tissue architecture and composition in *Flnb*^−/−^ cultured spines. Both TGFβ and p38 signaling have been implicated in multiple forms of disc degeneration^71–73^. In addition to the IVD, TGFβ signaling is also a major regulator of proteoglycan secretion in several tissues^74–77^, and our organ culture data suggest that its role in regulating type II collagen secretion in the IVD is minimal, which is supported by a study by Hegewald et al.^78^. While both TGFβ and BMP signaling are increased in *Flnb*^−/−^ IVD tissues, our data demonstrate that BMP signaling is increased at the postreceptor level via p38 activation and Smad1 linker phosphorylation from TGFβ/ERK activation. Therefore, with BMP2 stimulation, both Smad1 and p38 activation is increased via BMP2-specific receptor activation. Treatment with Noggin, however, targets multiple BMP receptors at the ligand level while also blocking other BMP ligands, likely disrupting additional BMP pathway components. Our in vitro results indicate that Smad1 activation is increased in *Flnb*^−/−^ tissues due to increased Smad1 linker phosphorylation via TGFβ/ERK and the inability of FLNB to stabilize i-Smad/r-Smad interactions. Therefore, inhibiting BMP at the receptor level would be ineffective. Our in vivo and cultured spine model results show that activation of p38 plays a major role in regulating type II collagen secretion in the IVD. Our findings showed that increased Smad 3, ERK, and p38 phosphorylation are major instigators of the *Flnb*^−/−^ disc degeneration phenotype, and inhibition of these pathway components in a combinatorial manner effectively rescued the *Flnb*^−/−^ disc in culture. It is possible that differing concentrations between multiple inhibitors could have an effect on rescuing *Flnb*^−/−^ and certainly warrants further study. This and our data suggest that therapies directed at disc degeneration either in Mendelian disorders or in the aging disc will likely need to be more nuanced and need to include manipulation of both canonical and noncanonical pathways.

As cytoskeletal proteins, filamins have been shown to bind and modulate at least 90 cytosolic proteins^12,79^; thus, it is likely that loss of FLNB affects numerous cellular activities. We limited our study to the TGFβ and BMP signaling pathways and demonstrated that these pathways are major instigators of the disc degeneration phenotype in the model. However, other pathways may also be involved and should be further delineated to identify more therapeutic target possibilities. Additionally, although the adult IVD is avascular and therefore ideal for long-term ex vivo experiments, it would be important to determine the effects of the proposed treatment in an in vivo setting, particularly to determine the effects on other tissues, which would allow this treatment to move forward into the realm of human health. In summary, our study uncovered that loss of FLNB negatively impacts TGFβ and BMP signaling through its role in modulating inhibitory Smads and provides a Mendelian disease mouse model to study disc degeneration and progressive fusions, a common disease of aging, and suggests a more nuanced treatment strategy for disc degeneration.

## Methods

### Study design

The objective of this study was to delineate the mechanism by which filamin B regulates TGFβ/BMP signaling. Following this, we also sought to confirm our findings while also outlining a potential treatment method. For the mechanistic portion of the study, we delineated our sample sizes based on IVD tissue availability, as it is difficult to extract a significant amount of protein from a single mouse IVD. Therefore, although our IVD cell studies show an *n* of 3, that number represents a total of nine mice in which at least 6 IVDs were extracted from the thoracic region of the spine. For chondrocytes, we conducted a power analysis using data from our previous study and determined that to detect a minimal difference of 30% in signaling activation by Western blot analysis between WT and *Flnb*^−/−^ mice with 80% power, a group size of at least *n* = 6 mice/group was required for biological replicates. The mice used for these replicates were taken from multiple litters, and investigators were blinded to genotypes during Western blot analyses. For ex vivo treatments, we conducted a large number of treatment conditions; therefore, each treatment condition was limited to three biological replicates to limit the number of mice sacrificed.

### Generation of mice

Mice used in this study were generated as previously described^49^. Briefly, the gene-trap vector pGT0Lxf containing a β-gal neomycin cassette and a splice acceptor site was inserted into intron 3 of *Flnb*, causing a shortened fusion transcript and loss of protein expression. The vector was contained in the 129/Ola ES cell line (BayGenomics RRF239). C57BL/6 J blastocysts were microinjected, and chimeras were mated with C57BL/6 J mice to generate *Flnb*^*+/–*^ mice. The mice used in this study were maintained on a mixed 129/Ola;C57BL/6j genetic background.

UCLA follows the PHS Policy, which requires that all institutions base their animal care and use programs on the Guide for the Care and Use of Laboratory Animals and that euthanasia be consistent with the American Veterinary Medical Association (AVMA) Guidelines on Euthanasia. The animals were monitored at least once daily and once during the weekend by personnel associated with this proposal and by the veterinary and technical staff of the UCLA Department of Laboratory Animal Medicine. Fresh water and food were supplied for consumption as needed, and visibly unhealthy animals were removed immediately and euthanized by inhalation. Sentinel animals were used for detailed serological testing to ensure that the colony remained pathogen free.

### Plastic sections and Von Kossa stain

Bone samples were fixed in 70% ethanol, dehydrated and embedded undecalcified in methyl methacrylate. Five-micrometer sections were cut on a Microm microtome (Richard-Allan Scientific, Kalamazoo, MI, USA) and stained with 0.1% toluidine blue, pH 6.4.

For Von Kossa Stain: Methyl methacrylate was removed using 1:1 xylene and chloroform for 30 min with constant stirring. Sections were washed in xylene for 1 min, three times in 100% ethanol, two times in 70% ethanol, once in 50% ethanol, and once in distilled water for two minutes. Sections were incubated in 5% silver nitrate at room temperature under UV light for approximately 20 min and then rinsed in 5% sodium thiosulfate for 30 s. Sections were counterstained with 5% MacNeal’s tetrachrome for 45 min and rinsed in distilled water. Sections were then dehydrated via two changes of 95% ethanol for 2 min each and one change of 100% ethanol for 2 min and then mounted.

### Histological analyses and immunohistochemistry

Dissected tissues were fixed using 10% neutral buffered formalin and decalcified using immunocal decalcification solution for a time period depending on the age of the sample, which was followed by paraffin embedding. Sagittal sections at 5–10 μm were generated and stained with Alcian Blue/Nuclear Fast Red. Staining sections were selected from the midpoint of the spine, and each sample was repeated in at least three biological replicates. At least three technical replicates were performed for each biological replicate.

Alcian Blue/Nuclear Fast Red staining was performed as follows: sections were deparaffinized and rehydrated and then incubated in 3% acetic acid solution. The sections were then stained in 10% Alcian Blue (Sigma, A5268)/3% acetic acid solution. Counter staining was performed using a 0.1% Nuclear Fast Red (Sigma, N8002)/5% Aluminum Sulfate (Sigma, A7523) solution.

For IHC, we boiled paraffinized and mounted sections for 20 min in Antigen Unmasking Solution (Vector). The sections were then stained via a Rabbit Specific HRP/DAB (ABC) Detection IHC Kit (Abcam). At least three biological replicates were used for all experiments, and staining of four sections per biological replicate was performed. Primary Antibodies used for IHC: Collagen II (Abcam, 34712, 1:50).

### Cell culture and tissue extraction

IVD discs used for the experiments were dissected from P15 mouse spines using a dissecting scope in a sterile phosphate buffered saline (PBS) bath. Following removal from the spine, IVDs were incubated at room temperature in DMEM + 10% FBS (Gibco) until we completed our dissections. Discs were then transferred to 10 mL of DMEM + 0.03% Type II Collagenase (Life Technologies) and incubated in a 37 °C/5% CO_2_ incubator overnight. Following neutralization of the collagenase solution using DMEM + 10% FBS, the cells were centrifuged at 1 000 r·min^−1^ for 7 min, plated, and incubated in DMEM + 10% FBS. For protein lysates, following centrifugation, the cell pellet was resuspended in RIPA buffer supplemented with phosphatase inhibitors (Sigma, P0044) and protease inhibitors (Sigma, P8340). For protein analyses, discs T5 through T11 were used.

Mouse primary chondrocytes were isolated from P1 mouse ribcages. Cleaned ribcage tissues were digested in 1 mg·mL^−1^ type II collagenase (Life Technologies)/serum-free DMEM solution (Gibco) in a 37 °C/5% CO_2_ incubator for 2 h. The isolated cells were then filtered through a 40 µm sterile cell strainer and plated in DMEM + 10% FBS (Gibco). Stimulation of primary chondrocytes and AF cells was performed at multiple time points with recombinant human TGFβ-1 (R&D, 10 ng·mL^−1^) ligand. The cells were then lysed in RIPA buffer supplemented with phosphatase and protease inhibitors. For the stimulation studies, six biological replicates were performed. The Subcellular Protein Fractionation Kit for Cultured Cells (Thermo Scientific) was used to extract cytoplasmic and nuclear fractions from primary chondrocytes. Three biological replicates were performed for these experiments.

MEFs were isolated from E13.5 mouse embryos. The heads were cut off and embryos were eviscerated. Embryos were minced, 1 mL of trypsin was added, and the mixture was incubated at 37 °C/5% CO_2_ for 30–45 min. Trypsin activity was quenched by adding 4 mL DMEM + P/S + 10% FBS media and pipetting 10–20X to break up tissues. The cellular suspension was passed through a 40 µm cell strainer and transferred to a T75 flask with 10 mL of media.

### MEF transfection and immunoprecipitation

MEFs were transfected with FLNB-GFP plasmid^80^, Smad 1-Flag (Addgene 12622), Smad 6-HA (Addgene 14962), and Smad 7-HA (Addgene 11733) using the Neon Electroporation Transfection System (Thermo-Fisher). MEFs were electroporated with 6 µg of plasmid, pulsed at 1 650 volts for 20 milliseconds and plated at 1.5 million cells per 10 cm plate.

Immunoprecipitation was completed as previously described^81^. Antibodies and reagents used: Anti-Flag antibody 4 µg (Sigma F7425), Anti-HA antibody 4 µg (Covance 902301), Anti-GFP antibody 5 µg (Abcam ab290), Protein A/G PLUS-Agarose Immunoprecipitation Reagent (Santa Cruz 2003).

### Western blot analysis

Protein lysates were generated by lysing cells in RIPA buffer supplemented with phosphatase and protease inhibitors (Sigma, P0044; Sigma, P8340). Isolated AF cells and primary chondrocyte lysates were quantified using a BCA protein quantification assay, and approximately 20 μg of protein in each well was separated by electrophoresis on 10% SDS-polyacrylamide gels with PVDF membranes used for transfer. Blocking was performed for 1 h in 5% milk/Tris-buffered saline-Tween (TBST), and membranes were incubated in 3% BSA/TBST solution with primary antibodies at 4 °C overnight. Species-specific horseradish peroxidase-conjugated secondary antibodies were used at a concentration of 1:2 000. Membranes were incubated for 1 h at room temperature. Signal detection was performed with the ECL plus kit (Cell Signaling, 7071). Band intensities were captured using a digital image scanner and were quantified if proven to be in the linear range. Quantification was performed using ImageJ (NIH, Bethesda, MD).

Primary antibodies used for Western blotting were as follows: phospho-Smad 3 (Cell Signaling, cs 9520, 1:1 000), Smad 3 (Cell Signaling 9523, 1:1 000), phospho-Smad 1/5/8 (Cell Signaling, cs 9511, 1:1 000), Smad 1 (Cell Signaling, 9743, 1:1 000), phospho-Erk p44/42 MAPK (Cell Signaling, cs 9101, 1:1 000), Erk p44/42 MAPK (Cell Signaling 9102, 1:1 000), phospho-p38 (Cell Signaling, cs 9211, 1:1 000), GAPDH (Cell Signaling, cs 2118, 1:1 000), and histone H3 (Abcam, 1:1 000).

### Organ culture

Spines containing multiple thoracic vertebral bodies and IVDs (T2-T11) were dissected from P1 mice and incubated in 24-well plates containing DMEM, 10% FBS, 100 U·mL^−^^1^ penicillin, 100 μg·mL^−^^1^ streptomycin, 50 μg·mL^−1^ ascorbic acid, and 1 μmol·L^−1^ beta-glycerophosphate. Spines were cultured for 12 days; media and inhibitors/ligands were changed every 48 h. The activity of each individual inhibitor is summarized in Table 1.

### Statistical analysis

GraphPad Prism was used for statistical analysis. Stimulated primary chondrocyte experiments were repeated with at least *n* = 6 biological replicates. To obtain enough protein, protein lysates generated from whole IVDs contained pooled IVD tissue samples derived from three different mice; from each of these three mice, six IVDs were taken. Therefore, a total of 9 mice were used in the IVD lysate experiments. Western blot band quantifications were normalized to GAPDH housekeeping gene levels. Each biological replicate was run on a separate Western blot. Therefore, mutant samples were quantified and evaluated as ratios against WT samples, and the WT samples were set to a value of one in the displayed data. All data points represent biological replicates of *Flnb*^−/−^ protein lysates, each normalized to a *Flnb*^+/+^ control. The dotted blue line in each graph represents the *Flnb*^+/+^ protein level, which is set to one upon normalization. Data were analyzed by two-sided Student’s *t* test; the results are shown as the mean ± interquartile range of the n number of trials reported in the figure legends. *P* ≤ 0.05 was considered statistically significant. Shapiro Wilks tests were performed to confirm the normal distribution of the results.

## Supplementary information

Supplemental Material

Supplementary figure 1

Supplementary figure 2

## Data Availability

All data associated with this study are present in the paper or the Supplementary Materials. Please contact DK (dkrakow@mednet.ucla.edu) for reagents and resources generated in this study.
