# Supplementary material for: Intervertebral disc degeneration is rescued by TGFβ/BMP signaling modulation in an ex vivo filamin B mouse model
Source: Bone Res. 2022 Apr 26;10:37. doi: 10.1038/s41413-022-00200-5 (PMC9042866; doi:10.1038/s41413-022-00200-5)
Supplement: Supplementary file 1 — Supplemental Material [file 41413_2022_200_MOESM1_ESM.docx]

**Supplemental Material**

**
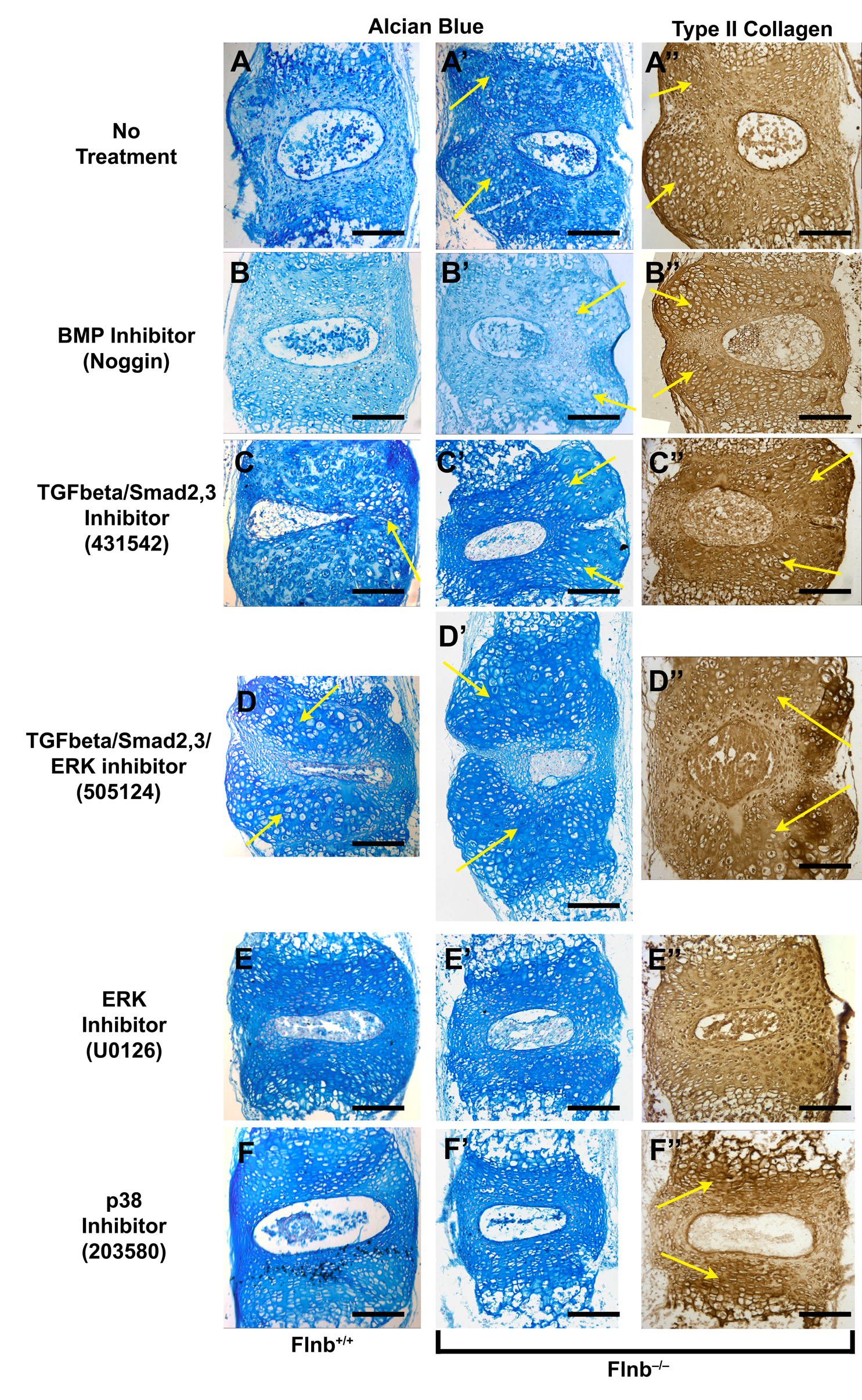
**

**Fig. S1. Inhibition of individual pathway components do not rescue *Flnb^–/–^* IVD phenotype.** (A-F) Sagittal sections of cultured IVDs stained with Alcian Blue for proteoglycan secretion or IHC-HRP using an antibody against Type II Collagen. (B-D) TGFβ and BMP inhibitors do not repair tissue architecture, proteoglycan, or type II collagen secretion in *Flnb^–/–^* IVDs. (E-F) Both ERK and p38 inhibitors repair tissue architecture but do not reduce proteoglycan secretion. ERK inhibition does not repair Type II Collagen secretion while p38 inhibition reduces Type II Collagen secretion in the AF while maintaining Type II Collagen levels in the AC (Yellow arrows). Yellow arrows indicate tissue architecture disruptions. N=3.

**
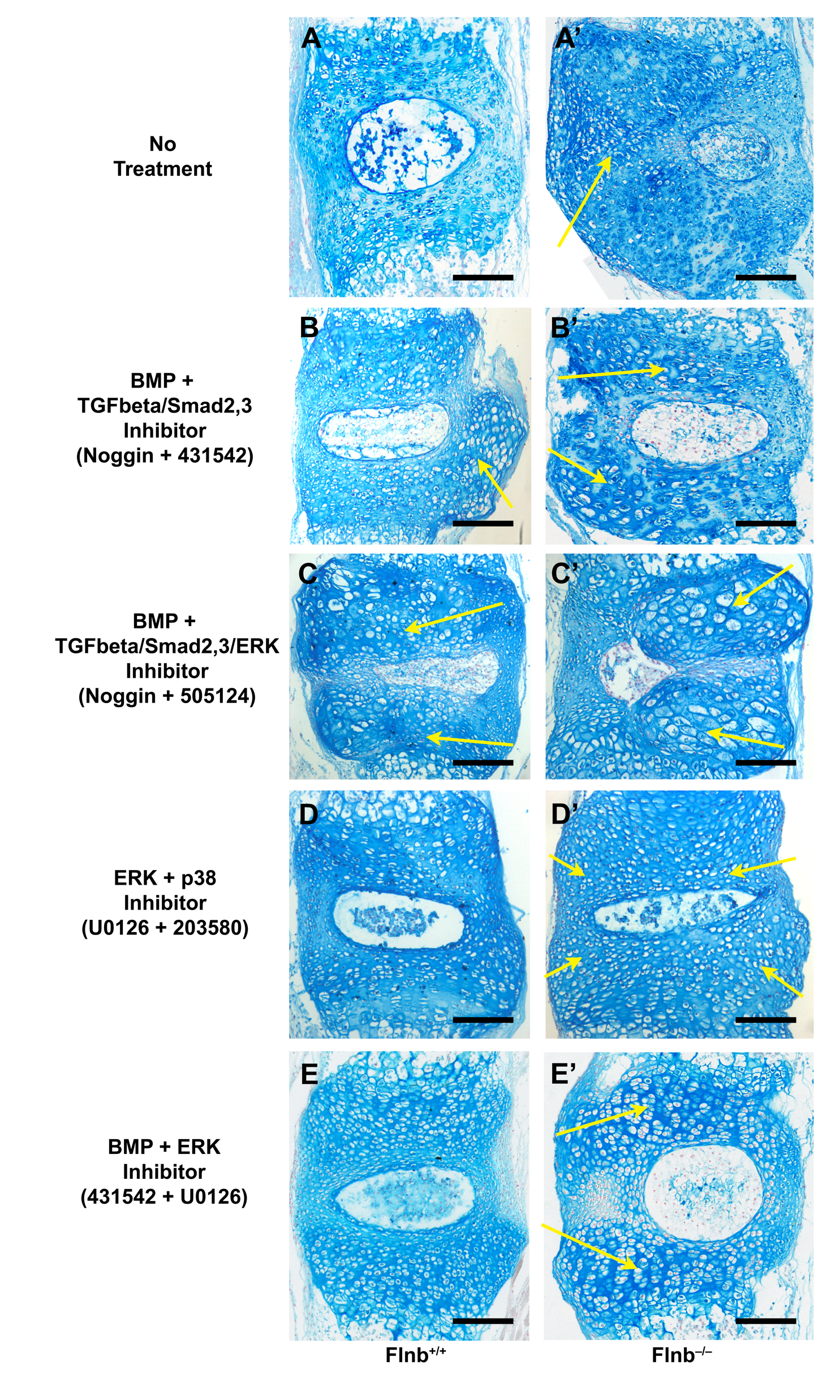
**

**Fig. S2. Inhibition of BMP and TGFβ in combination does not rescue *Flnb^–/–^* IVD phenotype.** (A-E) Sagittal sections of cultured IVDs stained with Alcian Blue for proteoglycan secretion. (B,B’) BMP and TGFβ inhibition does not improve proteoglycan levels and worsens tissue architecture in *Flnb^–/–^* IVDs. (D,D’) Inhibition of non-canonical pathways ERK and p38 results in more rounded cell morphology throughout the AF and proteoglycan levels are increased. (E,E’) Inhibition of BMP and ERK activation increases proteoglycan levels and worsens tissue architecture in *Flnb^–/–^* IVDs. Yellow arrows indicate tissue architecture disruptions. N=3.
