# Supplementary figures and images for: Intervertebral disc degeneration is rescued by TGFβ/BMP signaling modulation in an ex vivo filamin B mouse model
Source: Bone Res. 2022 Apr 26;10:37. doi: 10.1038/s41413-022-00200-5 (PMC9042866; doi:10.1038/s41413-022-00200-5)

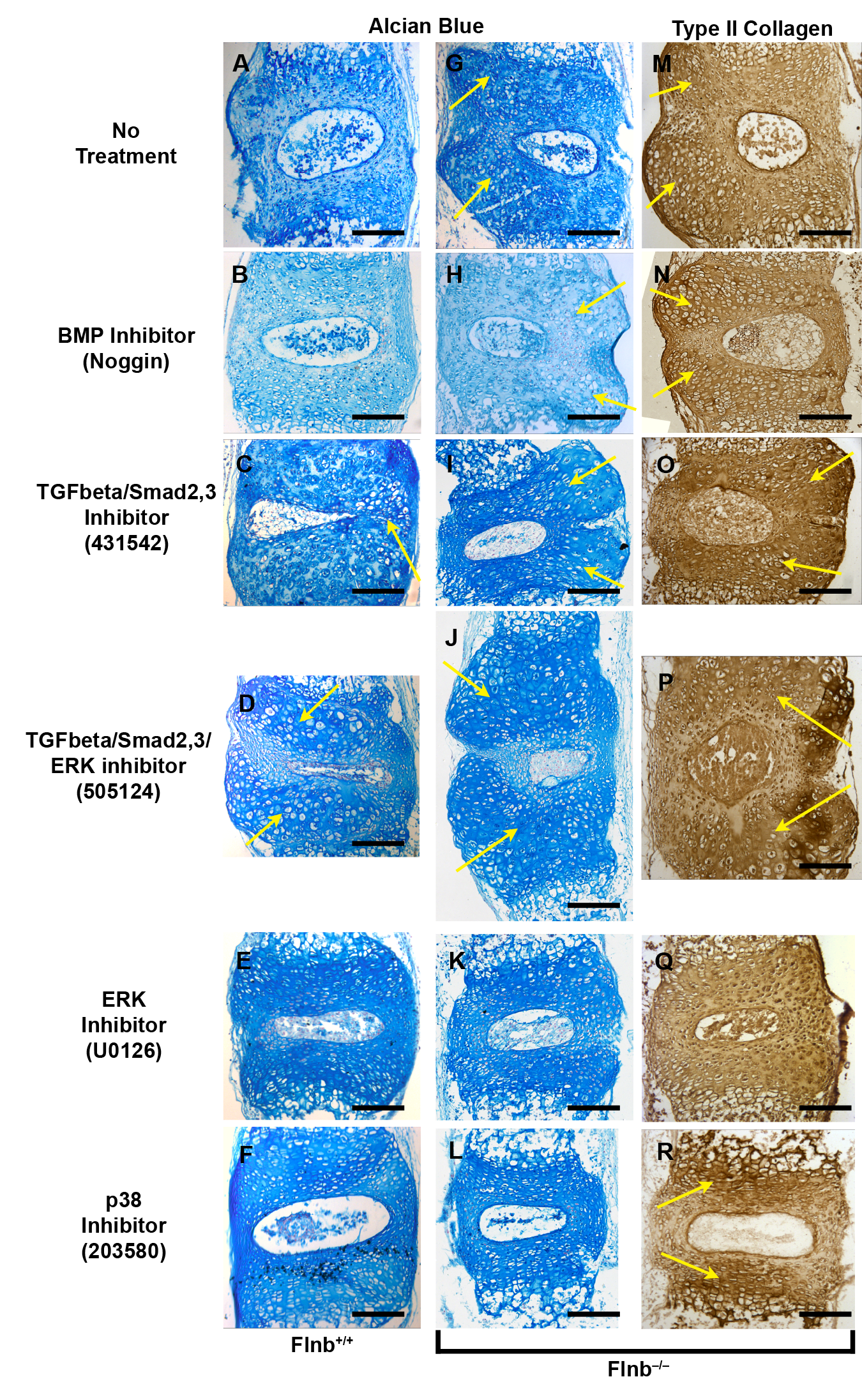

Supplement: Supplementary file 2 — Supplementary figure 1 [file 41413_2022_200_MOESM2_ESM.png]

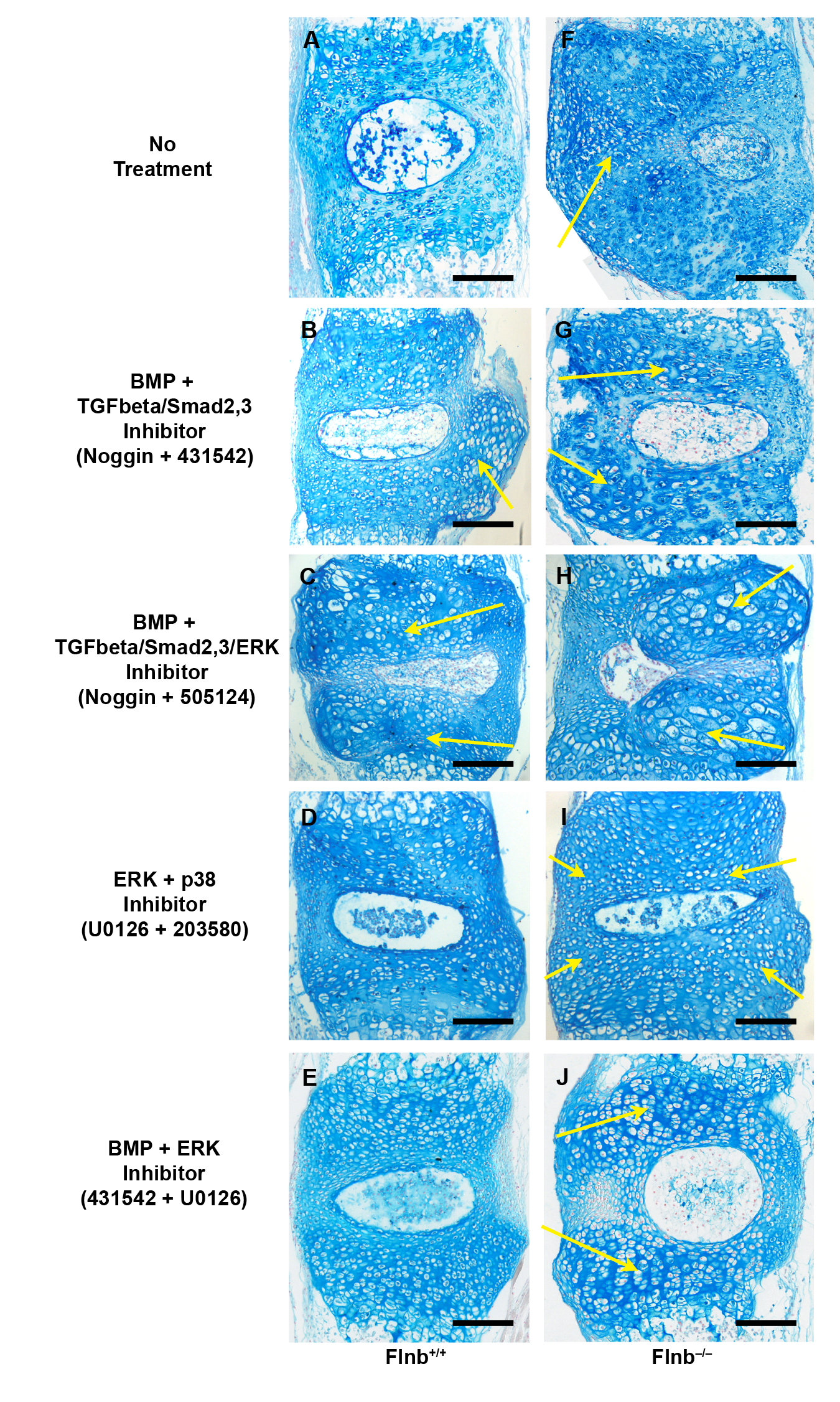

Supplement: Supplementary file 3 — Supplementary figure 2 [file 41413_2022_200_MOESM3_ESM.png]
